# Structural disconnectivity from paramagnetic rim lesions is related to disability in multiple sclerosis

**DOI:** 10.1002/brb3.2353

**Published:** 2021-09-08

**Authors:** Ceren Tozlu, Keith Jamison, Thanh Nguyen, Nicole Zinger, Ulrike Kaunzner, Sneha Pandya, Yi Wang, Susan Gauthier, Amy Kuceyeski

**Affiliations:** ^1^ Department of Radiology Weill Cornell Medicine New York New York USA; ^2^ Department of Neurology Weill Cornell Medicine New York New York USA; ^3^ Brain and Mind Research Institute Weill Cornell Medicine New York New York USA

**Keywords:** imaging biomarker, machine learning, multiple sclerosis, paramagnetic rim lesions, Quantitative Susceptibility Mapping, structural disconnectivity

## Abstract

**Background:**

In people with multiple sclerosis (pwMS), lesions with a hyperintense rim (rim+) on Quantitative Susceptibility Mapping (QSM) have been shown to have greater myelin damage compared to rim‐ lesions, but their association with disability has not yet been investigated. Furthermore, how QSM rim+ and rim‐ lesions differentially impact disability through their disruptions to structural connectivity has not been explored. We test the hypothesis that structural disconnectivity due to rim+ lesions is more predictive of disability compared to structural disconnectivity due to rim‐ lesions.

**Methods:**

Ninety‐six pwMS were included in our study. Individuals with Expanded Disability Status Scale (EDSS) <2 were considered to have lower disability (n = 59). For each gray matter region, a Change in Connectivity (ChaCo) score, that is, the percent of connecting streamlines also passing through a rim‐ or rim+ lesion, was computed. Adaptive Boosting was used to classify the pwMS into lower versus greater disability groups based on ChaCo scores from rim+ and rim‐ lesions. Classification performance was assessed using the area under ROC curve (AUC).

**Results:**

The model based on ChaCo from rim+ lesions outperformed the model based on ChaCo from rim‐ lesions (AUC = 0.67 vs 0.63, *p*‐value < .05). The left thalamus and left cerebellum were the most important regions in classifying pwMS into disability categories.

**Conclusion:**

rim+ lesions may be more influential on disability through their disruptions to the structural connectome than rim‐ lesions. This study provides a deeper understanding of how rim+ lesion location/size and resulting disruption to the structural connectome can contribute to MS‐related disability.

## Graphical abstract

The hyperintense rim lesions on Quantitative Susceptibility Mapping (QSM) (QSM rim+ lesions) have been shown to be particularly detrimental to tissue concentration causing greater myelin damage compared to chronic silent lesions (QSM rim‐ lesions) in multiple sclerosis (MS). However, there is no large study to date that has demonstrated the relationship of disability with rim+ lesions that are identified with QSM in MS. Our results showed that structural disconnectivity associated with rim+ lesions on QSM was more related to disability than structural disconnectivity associated with rim‐ lesions. Damages to the structural connections of the left cerebellum and thalamus from either lesion type were especially impactful on disability. This analysis provides a deeper understanding of how different lesion types can disrupt the structural connectome and contribute to MS‐related disability. A deeper understanding of the role of the connectome in MS is needed if we are to gain a comprehensive view of the disease to ultimately improve clinical outcomes in people with MS.

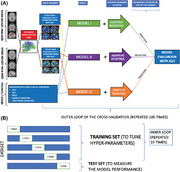

**Abbreviations**
ADA: adaptive boosting
AUC: area under curve
ChaCo: change in connectivity
EDSS: extended disability status score
QSM: Quantitative Susceptibility Mapping
MS: multiple sclerosis
NeMo: network modification
pwMS: people with multiple sclerosis
WM: white matter

## 1 INTRODUCTION

Multiple Sclerosis (MS) is a chronic disease characterized by inflammatory and demyelinating plaques within the central nervous system (CNS) (Weinshenker et al., 1991). Disability evolution is highly heterogeneous between people with MS (pwMS), making future prediction of disability progression very difficult (Barkhof, 2002; Pérez‐Miralles et al., 2013). Conventional magnetic resonance imaging (MRI) techniques are highly sensitive in detecting white matter (WM) lesions in pwMS, however, the correlation between lesion load measured with T2 imaging and clinical impairment is still modest (Filippi et al., 1995). This mismatch between traditional imaging biomarkers and clinical symptoms is known as the clinico‐radiological paradox in MS (Barkhof, 2002; Li et al., 2006). Advanced imaging techniques like Quantitative Susceptibility Mapping (QSM) (De Rochefort et al., 2010; Deh et al., 2015) may provide more information about lesion pathology (Wisnieff et al., 2015), that, in turn, may improve the understanding of the clinical implications of MS lesions (Chen et al., 2014; Kaunzner et al., 2019; Yao et al., 2018; Zhang et al., 2019).

QSM has been found to be more sensitive than conventional *T*2‐ and *T*2*‐weighted imaging as well as R2* ( = 1/*T*2*) mapping in the detection of iron accumulation in the brain (Cronin et al., 2016; Deistung et al., 2013; Langkammer et al., 2013; Stüber et al., 2016). Iron concentration in gray matter can differently relate to clinical disability. For example, higher iron concentration in the globus pallidus was related to more disability, while lower iron concentration in the thalamus was related to lower disability in pwMS (Zivadinov et al., 2018). Iron accumulation has also been identified in some WM lesions in pwMS (Zhang et al., 2019). Furthermore, WM lesions with a hyperintense rim appearance on QSM have increased inflammation on PK11195‐PET, a finding which was histopathologically confirmed by the presence of inflammatory cells in the rim, as well as larger volume and more myelin damage compared to QSM rim‐ lesions (Kaunzner et al., 2019; Yao et al., 2018). In rim+ lesions, greater myelin damage has been found, possibly due to higher iron accumulation, activated microglia and macrophages, and/or inflammation (Yao et al., 2018). It has been also shown that lesions characterized as rim+ are more likely to be slowly expanding and may persist for years, compared to rim‐ lesions which tend to shrink more quickly over time (Dal‐Bianco et al., 2017). Furthermore, the number of paramagnetic rim lesions as identified on phase MRI has been cross‐sectionally associated with worse disability (Absinta et al., 2019). However, no study to date has investigated the relationship between disability and QSM rim+ lesions in MS.

In addition to the differential pathology of the lesion, the clinical impact of a lesion is also related to its size and location (Charil et al., 2003; Krieger & Lublin, 2018) and subsequent impact on the structural and functional connectivity networks. In the past years, structural and functional connectome disruptions have been related to motor and cognitive dysfunction and depression in pwMS (Ashton et al., 2020; Ceccarelli et al., 2010; Dineen et al., 2009; He et al., 2009; Kuceyeski et al., 2018; Li et al., 2013; Llufriu et al., 2012; Nigro et al., 2015; Pagani et al., 2019). One way to investigate structural network disruptions of lesions is with the Network Modification (NeMo) Tool (Kuceyeski et al., 2013). The NeMo Tool uses a database of healthy tractograms on which the MS‐related lesion masks are superimposed to estimate the resulting regional disconnectivity pattern, that is, the percent of WM streamlines connecting to that region that also passes through a lesion. This approach has been used by our group and others to relate lesion‐related structural disconnectivity patterns to impairments, outcomes, functional connectivity disruptions, rehabilitation response, and gray matter pathology in pwMS (Fuchs, Carolus et al., 2018; Fuchs, Dwyer et al., 2018; Fusch et al., 2020; Kuceyeski et al., 2015, 2018).

To our knowledge, this is the first study that investigates the association between QSM rim+ lesions and disability in MS. The main aim of our study is to investigate how QSM rim+ and rim‐ lesions differentially impact structural connectivity and subsequent disability. Our hypothesis is that rim+ lesions’ disruption to the structural connectome will have a greater impact on disability. To test this hypothesis, we compared prediction accuracies of models classifying pwMS into disability categories using estimates of regional structural (WM) connectome disruption due to rim‐ and rim+ lesions. If rim+ lesions are more impactful in terms of clinical disability through their disruption of the structural connectome, then models based on these measures should perform better than the rim‐ lesions’ structural connectome disruption patterns. A secondary aim of this work was to identify which brain regions’ structural disconnections are most important in the classification of pwMS into disability categories. It must be noted that this type of approach does not consider the pathology type or severity of tissue damage within the lesion that may vary with lesion type—it only considers the lesion size and location and subsequent disruption of the structural connectivity network. If we can better understand how different lesion types can impact clinical outcomes through their disruption of the structural network, we may be able to better identify those patients at risk of disability and adjust treatments to minimize the burden of MS.

## 2 MATERIAL AND METHODS

### 2.1 Subjects

This is a cross‐sectional and retrospective study of patients with the diagnosis of MS meeting the 2010 McDonald criteria (Polman et al., 2011), age ≥ 18 years, and already participants within our research repository. Subject selection was based upon a random pull of data from 100 consecutive patients enrolled in the repository with QSM MRI data. From the 100 patients, 4 were not included given image quality and inability to accurately identify rim status of QSM lesions. This study consists of a cohort of 96 pwMS (age: 40.2 ± 10.1, 67% females) with a diagnosis of clinically isolated syndrome (CIS), relapsing‐remittent (RR), and primary progressive (PP) MS (CIS = 8, RR MS = 87, PP MS = 1). Demographic data were collected (age, sex, race, treatment duration, and disease duration), subjects underwent an MRI scan and Extended Disability Status Score (EDSS) was used to quantify disability. Treatment duration was computed as the duration between the start of first disease‐modifying treatment (pulled from the individual's medical record) until the date of MRI. PwMS were categorized into two groups: those with lower disability (n = 59 with EDSS <2) or those with greater disability (n = 37 with EDSS ≥ 2). This classification is based on EDSS values of 0–1.5 representing some abnormal signs but no functional disability appreciated. All studies were approved by an ethical standards committee on human experimentation, and written informed consent was obtained from all patients.

### 2.2 MRI data acquisition and processing

MRIs were acquired using a 3T GE scanner (Hdxt 16.0) with an eight‐channel phased‐array coil. Anatomical *T*1‐weighted sagittal 3D‐BRAVO (1.2 × 1.2 × 1.2 mm^3^), *T*2 (0.5 × 0.5 × 3 mm^3^), *T*2‐FLAIR (1.2 × 0.6 × 0.6 mm^3^) sequences were acquired. A QSM image was reconstructed from complex 3D‐GRE images (TR = 57 ms, first TE = 4.3 ms, echo spacing = 4.8 ms, number of echoes = 11, axial FOV = 24 cm, phase FOV factor = 0.8, acquisition matrix = 416 × 320 interpolated to 512 × 512, slice thickness = 3 mm, flip angle = 20°, bandwidth = 244 kHz, number of signal averages = 0.75, readout bandwidth = ±62.5 kHz) using a fully automated Morphology Enabled Dipole Inversion (MEDI + 0) method zero‐referenced to the ventricular cerebrospinal fluid (Liu et al., 2018; Spincemaille et al., 2019).

The conventional images (*T*1w, *T*2w, *T*2w FLAIR) were coregistered to the sum‐of‐squares echo‐combined magnitude GRE images using the FMRIB's Linear Image Registration Tool algorithm (Jenkinson et al., 2002); automated brain segmentation was performed using FreeSurfer (Fischl et al., 1999). WM tissue segmentations were manually edited for misclassification due to WM *T*1‐hypointensities associated with lesions. The WM hyperintensity lesion masks were created from the *T*2 FLAIR images by categorizing the tissue type based on the image intensities within the Lesion Segmentation Tool using the LPA method; the masks generated were further hand edited, if necessary. Next, the *T*2FLAIR lesions masks were coregistered to the QSM images and further hand edited (if needed) to better match the lesion geometry on QSM. A lesion was designated as rim+ if QSM was hyperintense at the edge of the lesion. Complete and partial hyperintense QSM rim lesions were considered to be rim+ lesions. The presence of a partial and/or full hyperintense QSM rim was determined by a trained neurologist (U.K.) and neuroradiologist (Weiyuan Huang). In the case of disagreement of two reviewers, an independent third neurologist (S.G.) decided on the presence of a positive hyperintense rim. Once the rim+ lesions were identified, they were removed from the T2FLAIR lesion masks to obtain a rim‐ lesion mask.

QSM rim+ lesion masks were transformed to the individual's *T*1 native space using the inverse of the *T*1 to GRE transform and nearest‐neighbor interpolation. Individual *T*1 images were then normalized to MNI space using FSL's linear (FLIRT) and nonlinear (FNIRT) transformation tools (http://www.fmrib.ox.ac.uk/fsl/index.html); transformations with nearest‐neighbor interpolation were then applied to transform both native anatomical space lesion masks to MNI space. The transformations were concatenated to minimize interpolation. Lesions were manually inspected after the transformation to MNI space to verify accuracy. The MNI space rim‐ and rim+ lesion masks were processed through the newest version of the NeMo Tool (Kuceyeski et al., 2013), NeMo Tool 2.0, that estimates the resulting pattern of structural disconnectivity due to a given lesion mask. NeMo Tool 2.0 calculates the Change in Connectivity (ChaCo) score for each of 86 cortical, subcortical, and cerebellar regions, which is defined as the percent of tractography streamlines connecting to that region that also pass through the lesion mask. The newest version of the tractography database consists of structural connectomes from 420 unrelated healthy controls (206 female, 214 male, 28.7 ± 3.7 years), see Supplemental Information for details on the creation of the tractography database. ChaCo scores were extracted separately from rim‐ and rim+ lesion masks. The ChaCo scores from the rim‐ lesion mask were computed across all subjects, while the ChaCo scores from rim+ lesion masks were computed only for the subjects who had at least one rim+ lesion (N = 56). The Chaco scores for the subjects without rim+ lesions were 0, since there was no structural disconnectivity due to rim+ lesions for these subjects. To test for differences in the regional disconnectivity patterns of rim+ and rim‐ lesions, a Wilcoxon rank‐sum test was performed on the regional ChaCo scores from the rim‐ and the rim+ lesion masks over the 56 pwMS that had at least one rim+ lesion. A Wilcoxon rank‐sum test was used to test for differences in regional structural disconnectivity between disability groups for both rim+ and rim‐ lesion ChaCo scores. Regions were considered significantly different if *p* < .05, after Benjamini–Hochberg (1995) correction for multiple comparisons.

### 2.3 Modeling and statistical analysis

Classification was performed using the Adaptive Boosting (ADA) method, a boosting algorithm of decision trees (Alfaro et al., 2013), see Supporting information for details. For the task of classifying pwMS into lower versus greater disability groups, three models were created based on demographic or clinical variables (age, sex, race, disease duration, and treatment duration): (i) ChaCo scores from rim‐ lesions (Model I), (ii) ChaCo scores from the rim+ lesions (Model II), (iii) both sets of ChaCo scores from rim‐ and rim+ lesions (Model III). All subjects were included in all models, where the ChaCo scores from rim+ lesions were considered as 0 for the pwMS without rim+ lesions (n = 40) as there was no structural disconnectivity due to rim+ lesions for these patients.

**FIGURE 1:**
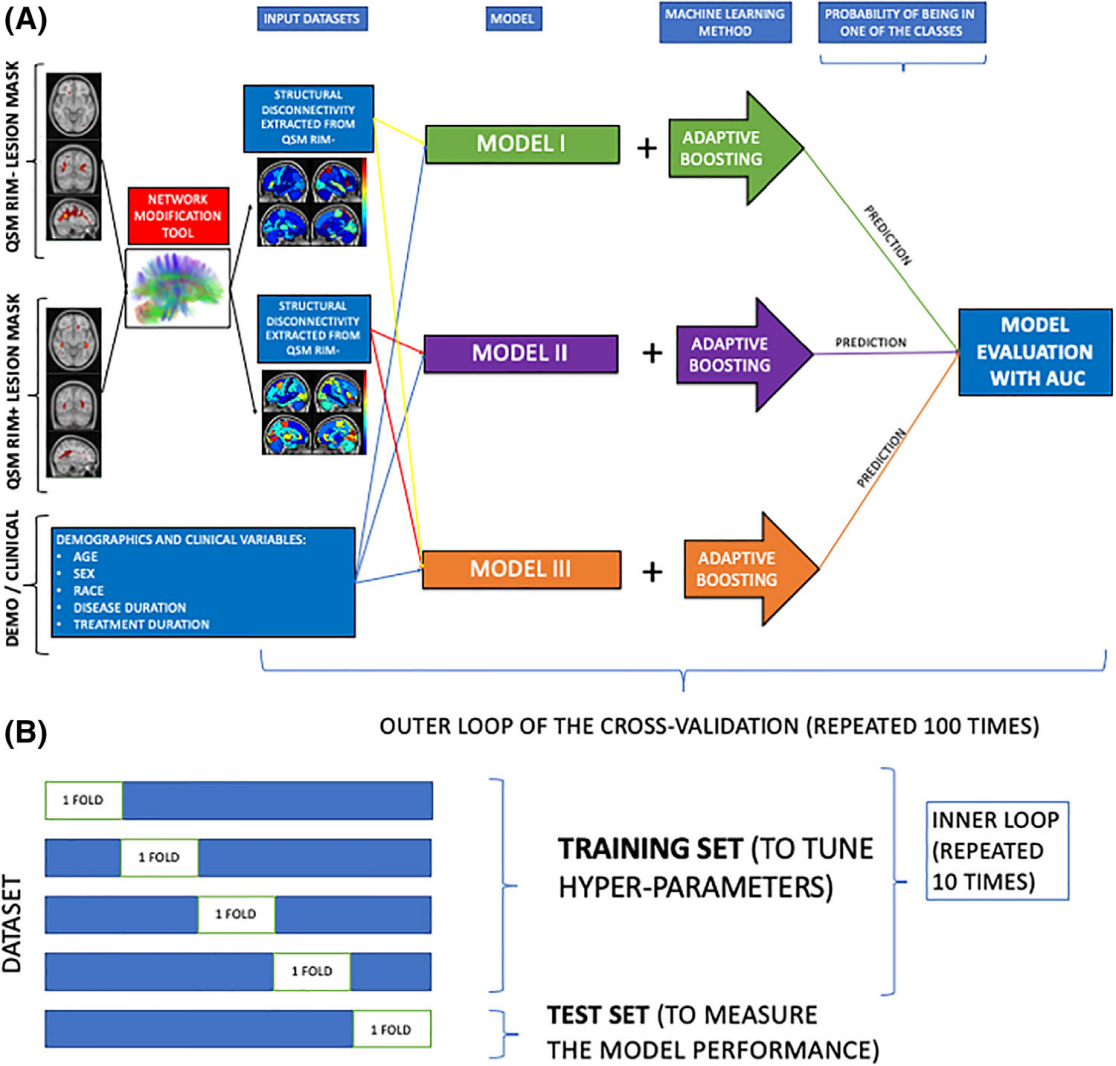
Overview of the analytic pipeline workflow, including (A) calculation of ChaCo from rim‐ (*T*2 FLAIR lesions without rim+ lesions) and rim+ lesion masks and ADA models, and (B) the cross‐validation scheme for the training and testing of the ADA models

The ADA model was trained with two cross‐validation loops to optimize the hyperparameters and build the model (fivefolds inner) and test the performance on hold‐out data (fivefolds outer), see Figure 1. The inner loop performed grid‐search to find the set of hyperparameters that maximized area under the Receiver Operating Characteristics curve (AUC) in the validation set. Synthetic Majority Over‐sampling Technique (SMOTE) (Chawla et al., 2002) was used to obtain a class‐balanced training dataset to improve the prediction accuracy for the minority class. SMOTE compensates for imbalanced classes by creating synthetic examples using nearest neighbor information instead of creating copies from the minority class, and has been shown to be among the most robust and accurate methods with which to control for imbalanced data (Santos et al., 2018). The inputs are standardized in the inner loop and in the outer loop to avoid data leakage. A final model was built using the entire training dataset with the optimal hyperparameters and assessed on the hold‐out test set from the outer loop. The outer loop was repeated using 100 different random partitions of the data. The average of AUC (over all fivefolds × 100 iterations = 500 test sets) was calculated to assess the performance of the models. Performance metrics for the three models were compared using permutation test of 1000 permutations (David, 2008). The models were considered significantly different when *p* < .05 (after Benjamini–Hochberg correction for multiple comparisons). The relative importance of the input variables in the final ADA models was calculated using the weight of the tree and gain of the Gini Index, which is given by a variable in a tree (Alfaro et al., 2013). The software R (https:/www.r‐project.org) version 3.4.4 and Matlab version R.2020a were used for all statistical analyses and graphs.

## 3 RESULTS

### 3.1 Characteristics of pwMS

**FIGURE 2:**
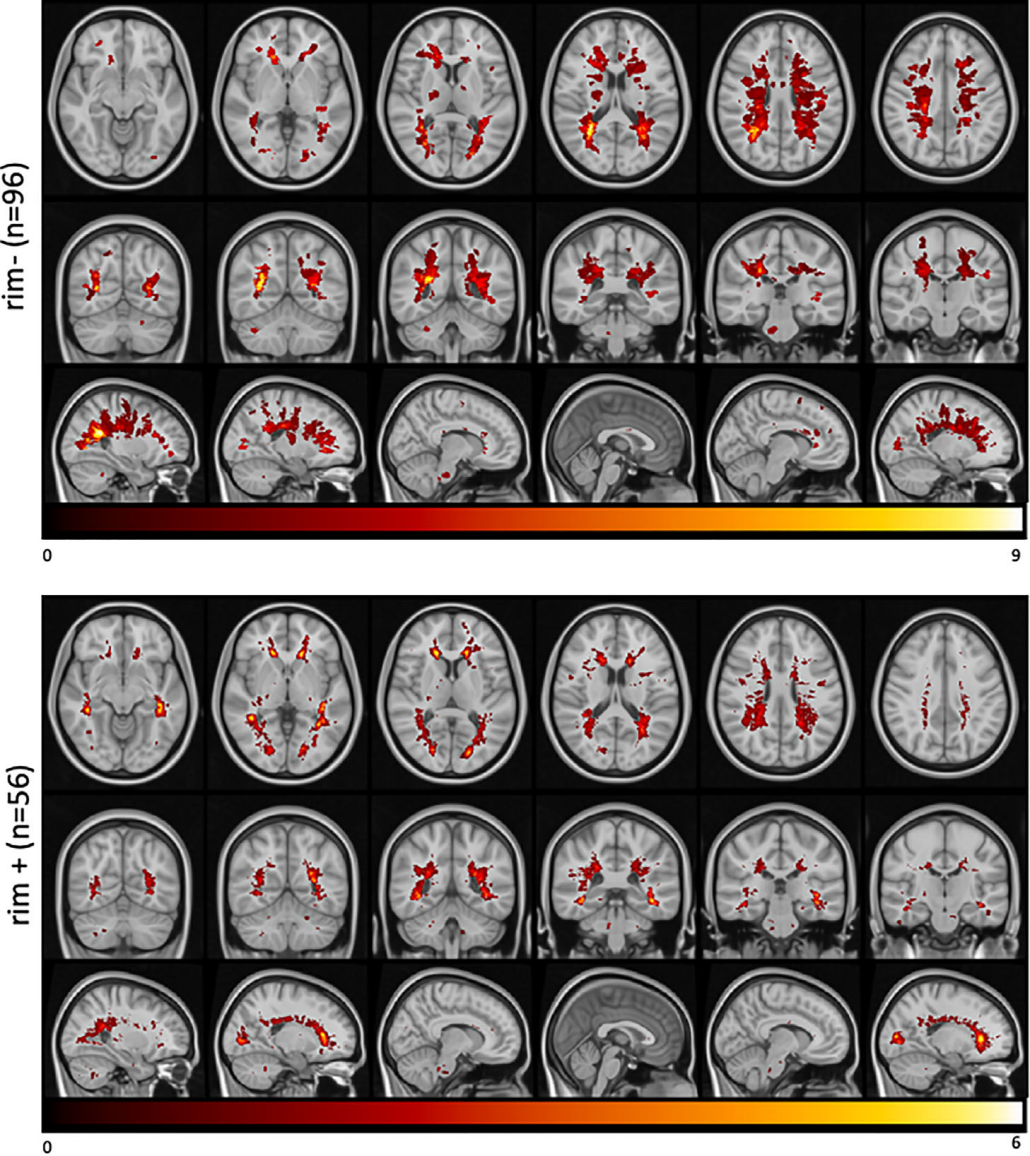
Heatmap of the lesion masks extracted from (A) rim‐ (*T*2 FLAIR lesions without rim+ lesions) and (B) rim+ images. Color indicates the number of individuals (out of 96 and 56, respectively) that had a lesion in that voxel

Table 1 displays subject demographics, clinical information, and the number of rim‐ and rim+ lesions. PwMS that had lower disability (N = 59) were significantly younger, had shorter disease duration, and significantly fewer rim‐ lesions than those with greater disability (N = 37) (*p*‐value < .05 for all comparisons). The ratio of rim+/rim‐ lesions was similar in the greater versus lower disability categories (0.05 vs. 0.03, *p*‐value = .81). A higher percent of those pwMS with at least one rim+ lesion had greater disability (43%) compared to the percent of pwMS with no rim+ lesions and greater disability (33%), see Supporting information Table S1. The demographics and clinical variables were also compared between pwMS with (n = 56) and without (n = 40) rim+ lesions, see Supporting information Table S2. There was no significant difference in demographics and clinical variables between these two groups (*p*‐value > .11 for all comparisons). The rim+ and rim‐ lesion volumes were compared in those 56 pwMS with rim+ lesions; rim+ lesions were significantly larger in volume (median 343 mm^3^, IQR: [173, 572]) than rim‐ lesions (median 69 mm^3^, IQR: [69, 145]). When the number of the lesions was analyzed within the pwMS with rim+ lesions (n = 56), the median of the rim ‐ lesions was 28.5, while the median of the rim+ lesions was 3.5. Heat maps of lesion masks for the two lesion types are shown in Figure 2, where it can be qualitatively appreciated that rim+ lesions tended to cluster in periventricular WM, compared to rim‐ lesions that are more widespread throughout the WM.

**TABLE 1:** Clinical, demographic, and imaging characteristics for all 96 pwMS (second column) and split into disability categories (third column)

	All (n = 96)	Lower disability (n = 59)	Greater disability (n = 37)	*p*‐value
Female%	67	68	65	.94
Race%	African American: 16 Asian: 2 Caucasian: 72 Hispanic: 5 Other: 5	African American: 11 Asian: 1 Caucasian: 76 Hispanic: 5 Other: 5	African American: 24 Asian: 2 Caucasian: 64 Hispanic: 2 Other: 5	.56
Age	38 [32, 48]	35 [30, 42]	47 [37, 51]	.0007
Disease duration	4.6 [2.5, 11.2]	4.1 [2.2, 8.3]	7.9 [3.2, 15.9]	.012
Treatment duration	3.1 [1.5, 6.1]	2.4 [1.3, 4.4]	3.7 [2.3, 8.0]	.06
EDSS	1 [0, 2]	0 [0, 1]	2.5 [2, 4]	<10^–16^
# of rim‐ lesions	19 [11, 44.25]	16 [8, 30]	39 [14, 59]	.006
# of rim+ lesions	1 [0, 4]	1 [0, 3]	2 [0, 5]	.15
% rim+ over all lesions	0.04 [0, 0.13]	0.03 [0, 0.16]	0.05 [0, 0.13]	.81

The *p*‐values that were obtained in comparison of lower versus greater disability groups were presented in the fourth column. Values are presented as median [1st quartile, 3rd quartile] for the continuous variables, *p*‐values are corrected with Benjamini–Hochberg method for multiple comparisons. Age, disease, and treatment duration were measured in years.

### 3.2 Structural disconnectivity from rim‐ and rim+ lesions

**FIGURE 3:**
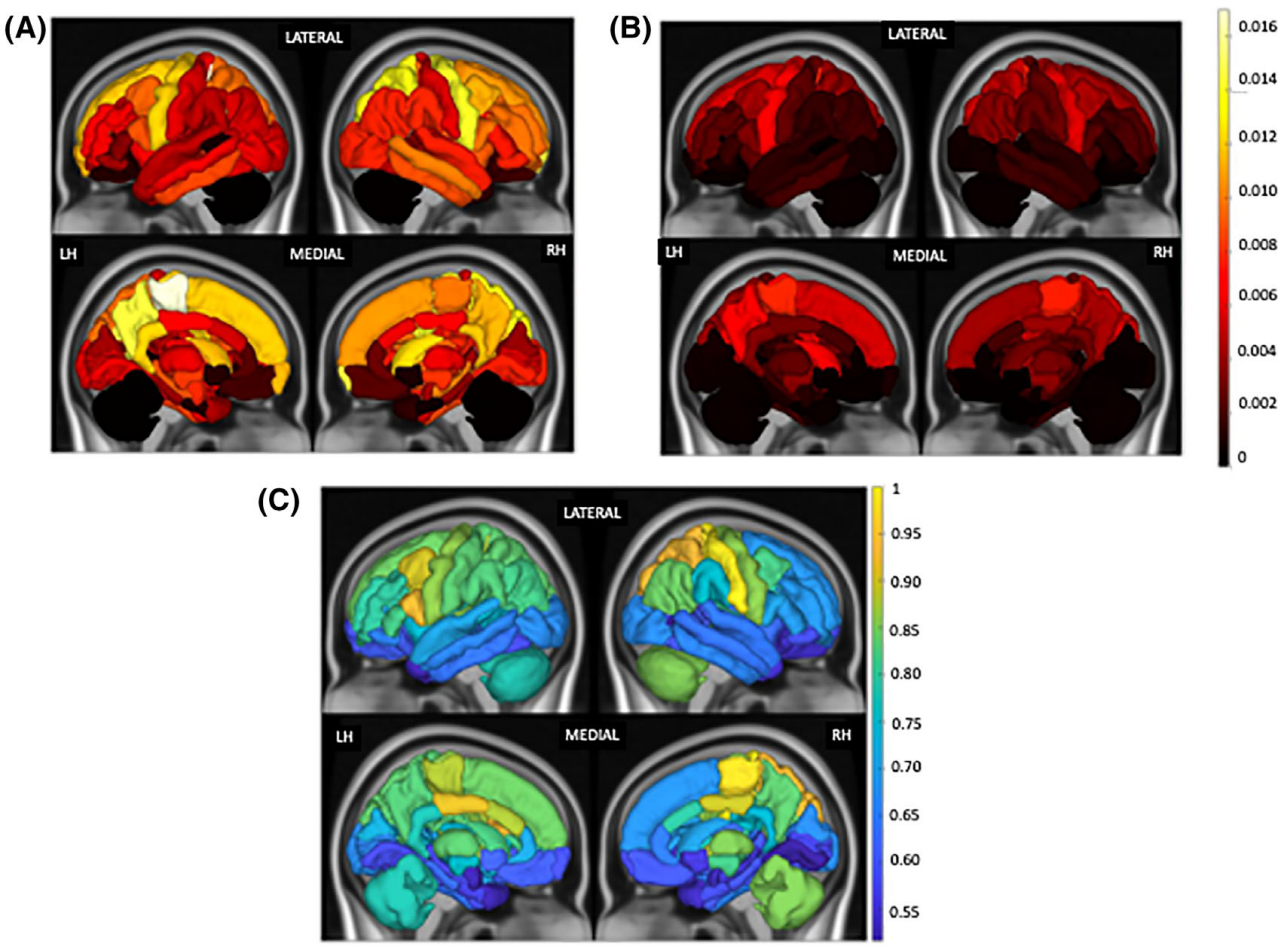
Median of ChaCo extracted from (A) rim‐ lesion mask (*T*2 FLAIR lesions excluding rim+ lesions) across all pwMS (N = 96) and (B) rim+ lesion masks, only for the pwMS who had at least one rim+ lesion (N = 56). The colorbar shows the ChaCo for the figure (A) and (B). (C) Relative paired Wilcoxon rank‐sum statistic (divided by maximum value) indicating all regions had greater ChaCo from rim‐ lesion masks than from rim+ lesion masks (considering only the 56 pwMS who had at least one rim+ lesion). Median ChaCo from rim+ lesion masks were computed only for the subjects who had rim+ lesions (N = 56), while the median ChaCo from the rim‐ lesion mask was computed across all subjects. Note the scale differences in the two modalities—this is mostly due to the fact that there were far fewer rim+ lesions than rim‐ lesions

Regional ChaCo scores of structural disconnectivity based on the rim‐ and rim+ lesion masks are visualized in Figure 3. Median ChaCo scores from rim+ lesion masks were computed only for the subjects who had rim+ lesions (N = 56), while the median ChaCo scores from the rim‐ lesion mask were computed across all subjects. Note the scale differences in the two modalities—this is mostly due to the fact that there were far fewer rim+ lesions than rim‐ lesions. Left paracentral, left precuneus, and bilateral precentral (primary motor) regions had highest disconnectivity in both the rim‐ and rim+ lesion masks. Right and left putamen also had higher disconnectivity from the rim‐ and rim+ lesion masks, respectively, compared to other regions. ChaCo scores from rim‐ lesion masks were significantly higher than those from rim+ lesion mask for all regions (*p* < .05, corrected), particularly, in right primary motor and right paracentral gyrus.

### 3.3 Structural disconnectivity differences across disability subgroups

**FIGURE 4:**
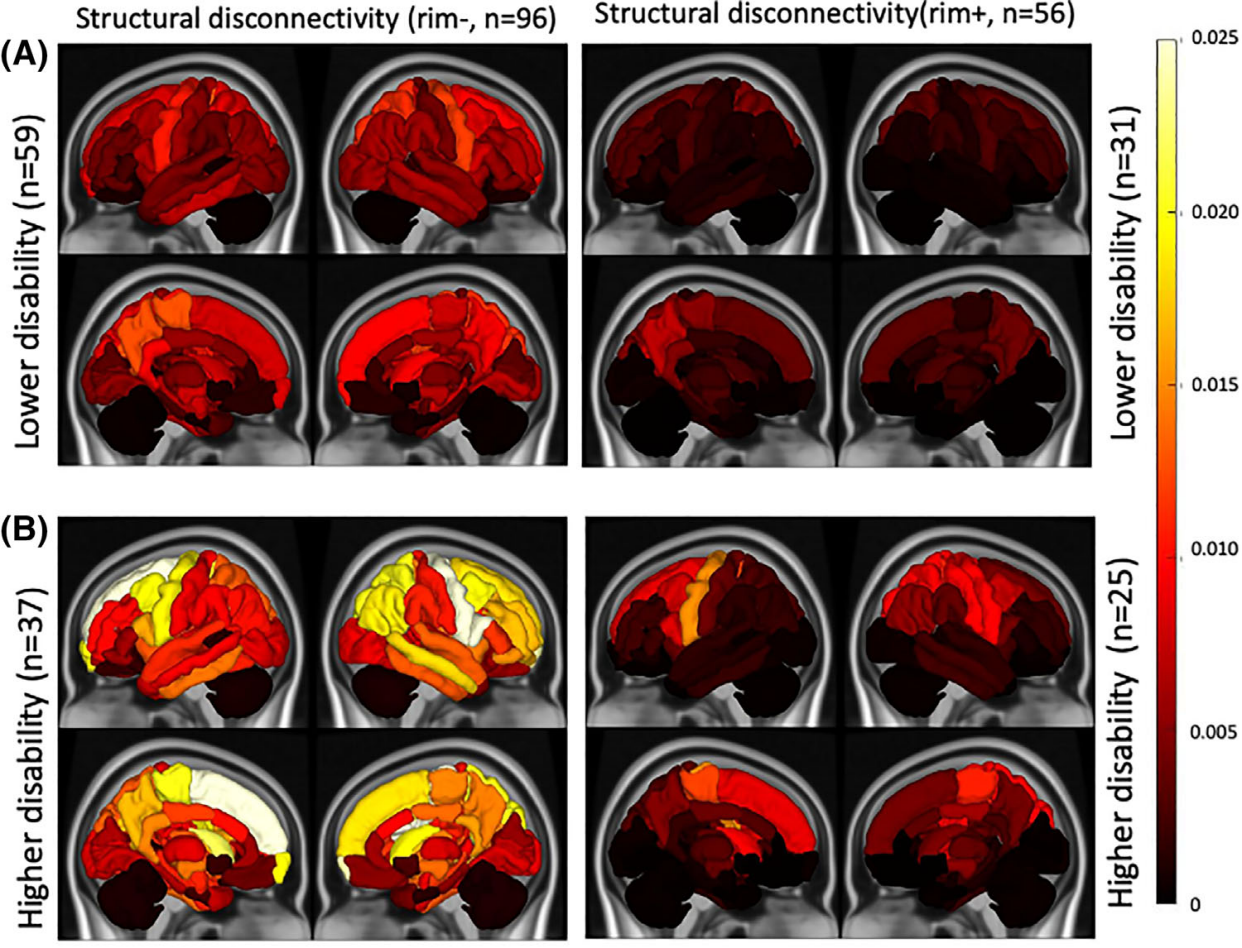
Median of ChaCo extracted from rim‐ (*T*2 FLAIR lesions excluding rim+ lesions) and rim+ lesion masks for pwMS (A) lower disability versus (B) those with greater disability

Figure 4 illustrates the median Chaco scores for each subgroup of categories, that is, lower versus greater disability for the two lesion types. The ChaCo scores based on rim‐ lesion masks were significantly larger in 22 regions, most prominently in the left frontal areas, in the pwMS with greater disability compared to those with lower disability (*p* < .05, corrected), see Supporting information Figure S1. There were no significant differences between the disability subgroups for the ChaCo scores based on rim+ lesion masks.

### 3.4 Classification results

**FIGURE 5:**
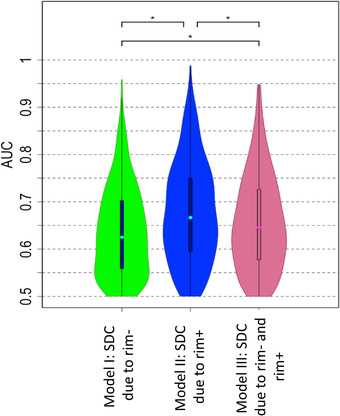
AUC results obtained with Model I (ChaCo from rim‐ lesion masks), Model II (ChaCo from rim+ lesion masks), and Model III (both rim‐ and rim+ lesion ChaCo) for the classification task of lower versus greater disability. AUC results were obtained over the 100 outer loops and 5 test datasets for each outer loop for the disability classification task. *indicates significant differences in AUC, corrected *p* < .05

Figure 5 depicts the three models’ distributions of AUC over the 100 outer loops and five test datasets for each outer loop for the disability classification task. Model II, which included demographics/clinical variables and ChaCo scores from the rim+ lesions, had significantly higher AUC (median of 0.66) than Model I (median of 0.62) or Model III (median of 0.64) (corrected *p*‐values < .007 for all comparisons). Supporting information Figure S2 illustrates balanced accuracy, sensitivity, and specificity for the three models for comparison to previous findings. Model II had significantly higher sensitivity, specificity, and balanced accuracy than Models I and III (*p*‐value < .05 for all, corrected), while there was no difference in these metrics between Models I and III (*p*‐value = .47 for sensitivity, .37 for specificity, and .38 for balanced accuracy). The classification models were rerun using only CIS and RRMS patients (i.e., one pwMS with PPMS was excluded as their disease mechanisms or progression may be different from other subtypes). The results were similar to what we observed with the original dataset: Model II performed significantly better than Models I and III (*p* < .05). However, there was no significant difference between Models I and III (*p*‐value = .13).

### 3.5 Variable importance

**FIGURE 6:**
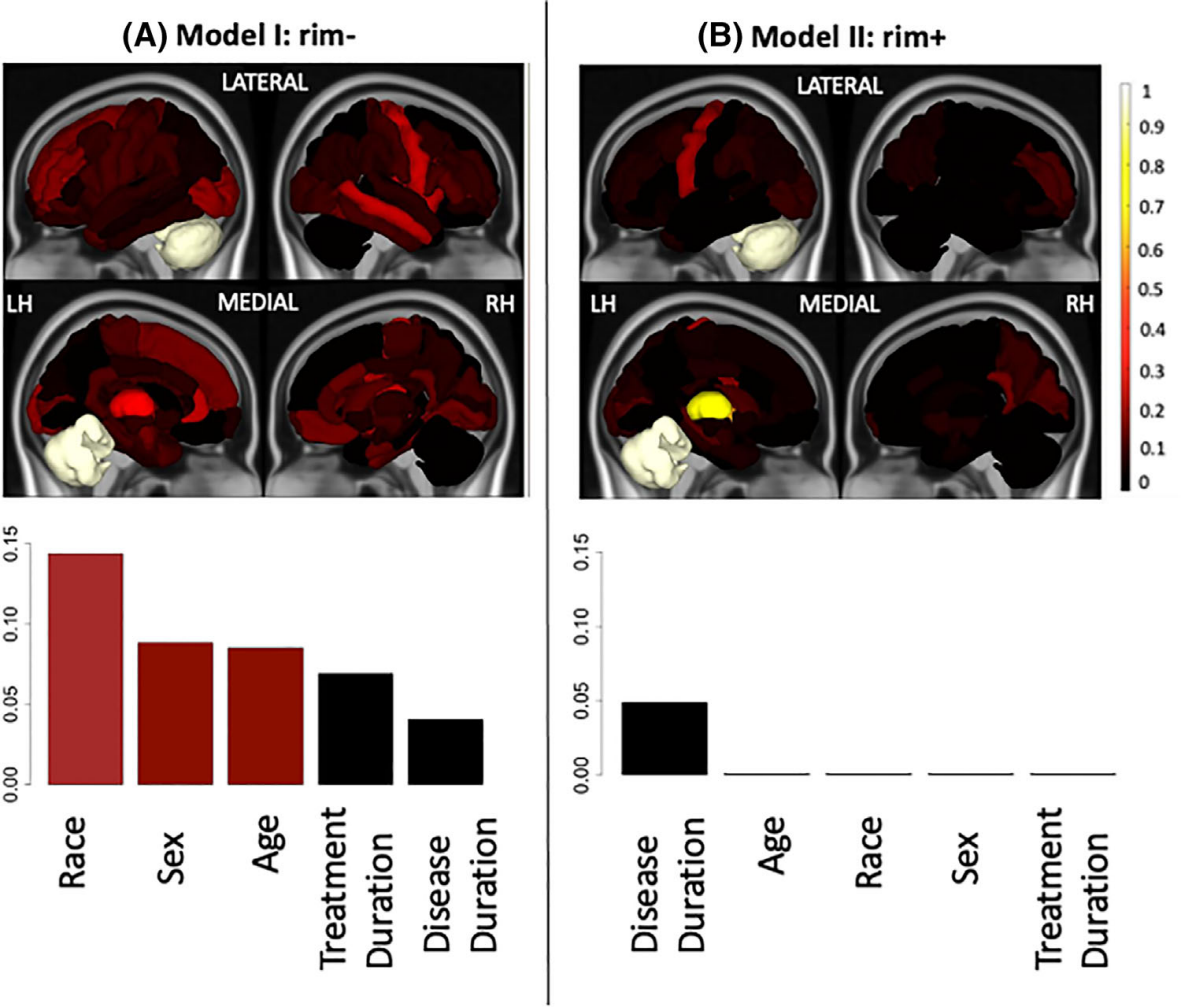
Relative feature importance for the models that included demographics and regional ChaCo due to (A) Model I: rim‐ lesions (T2 FLAIR lesions excluding rim+ lesions) (left column) and (B) Model II: rim+ lesions (right column) for the classification of pwMS with greater disability versus those with lower disability. Feature importance for the regional ChaCo scores are visualized via brain volumes and demographic variable importance by bar plots. Third quantiles of the feature importance distributions are visualized due to the distribution skewness. Relative importance values for all figures were obtained by dividing that variable's feature importance by the maximum importance value across both models

Figure 6 shows the feature importance of the structural disconnectivity measures (ChaCo scores) and demographics/clinical variables for Models I and II in classifying disability. The third quartiles of the feature importance scores were visualized since the data were highly skewed. Structural disconnection in the left cerebellum and left thalamus due to both rim‐ and rim+ lesions were the most important variables in classifying disability groups. Race, sex, and age were important in Model I and disease duration was important in Model II. Race and disease duration were ranked as the 17th and 22th most important variables among 86 imaging and 5 demographics/clinical variables in Models I and II, respectively.

## 4 DISCUSSION

In this study, we investigated the patterns of structural connectome disruption arising from two types of lesions that occur in pwMS, namely those with a hyperintense rim on QSM (rim+) and those without. We compared the classification accuracy of models using the structural disconnectivity of the two lesion types to predict disability categories in pwMS. Our main findings were that (1) pwMS had high structural disconnectivity in motor regions (precentral and paracentral gyri) resulting from both types of lesions, (2) rim+ lesions were larger than rim‐ lesions, tended to more frequently occur in periventricular areas and, thus, impact structural connectivity disproportionately in periventricular regions, (3) structural disconnectivity from rim+ lesions better classified pwMS into disability categories than structural disconnectivity from rim‐ lesions, and (4) structural disconnectivity in left cerebellum and left thalamus resulting from both lesion types were among the most important features in the classification of disability categories.

### 4.1 Structural disconnectivity from MS lesions is highest in motor regions and rim+ lesions are more periventricular than rim‐ lesions

The regions with the highest structural disconnection scores from both types of lesions were mostly motor‐related, including paracentral, precentral, and putamen. It appears from Figures 2 and 3 that, in addition to the rim‐ lesions being more numerous and, thus, having larger disconnectivity measures, the two lesion types tend to have slightly different spatial locations and regional patterns of structural disconnectivity. The rim‐ lesions appear to be distributed widely throughout the WM while the rim+ lesions tend to cluster around the ventricles. The ChaCo scores reflect that most of the regions with disconnection from the rim+ lesions are indeed periventricular and the remainder of the brain is relatively spared; this stands in contrast to the ChaCo scores from rim‐ lesions that are more widespread across the brain.

### 4.2 Structural disconnectivity from rim+ lesions better predicts disability category in pwMS

Previous cross‐sectional studies classifying pwMS into disability categories based on imaging biomarkers, including connectome measures, have shown similar prediction performance with an accuracy and AUC between 0.50 and 0.67 (Zhong et al., 2017; Zurita et al., 2018). The relationship between connectome disruption due to *T*2FLAIR lesions and cross‐sectional and longitudinal disability change, such as processing speed deficits, has been previously assessed (Kuceyeski et al., 2015, 2018). Even though the difference in AUC results from rim‐ and rim+ models was modest, our findings suggest that rim+ lesions also may be slightly more influential on disability compared to rim‐ lesions through their specific patterns of disruption to the structural connectome. We conjecture that structural disconnectivity due to rim+ lesions may be more detrimental due to their (1) much larger volume, as seen in this and other work (Zhang et al., 2019), and (2) periventricular location, which may result in damage to WM that is more central in the brain and, thus, more disruptive to the overall network structure. It must be emphasized that only the structural disconnectivity due to the lesion, which is influenced by its size and location, was used in our classification models of disability. No explicit information about the microstructural tissue pathology severity (such as iron concentration, demyelination, axonal loss, presence of inflammation, edema, etc.) was included in the calculation of the ChaCo scores or as variables in the predictive model. If a rim+ lesion and rim‐ lesion were the same exact size and location, they would have the same ChaCo score (albeit for different lesion mask types), even if the rim+ lesions were more damaging to WM microstructure. Therefore, when we say that the model based on rim+ lesions may be more informative of disability, the conclusion can be stated only for the pattern of structural disconnection due to the rim+ lesion size/location relative to the pattern of structural disconnection due to rim‐ size/location. This is an interesting finding in and of itself—that despite the lack of explicit pathology information, the size and location of the rim+ lesions do seem to be more informative of disability status than the rim‐ lesions. If it so happens that rim+ lesions have more inflammation, iron deposition and/or demyelination, then there may be some gross pathological information contained implicitly in the separation of the lesion types and subsequent ChaCo score calculation; however, we do not think our results can be used to make statements about the microstructural or pathological differences that may exist between the two lesion types that may have differential impact on WM anatomical or physiological health. The model that included the disconnectivity due to both rim+ and rim‐ lesions performed significantly worse than the model including structural disconnectivity due to rim+ lesions only. This is likely due to the increase in dimensionality (×2) of the number of variables in the input data which can cause model overfitting.

The classification models were rerun using only 56 pwMS who had at least one rim+ lesions. Even though the models including structural disconnectivity due to rim+ lesions gave slightly better AUC results than the model including structural disconnectivity due to rim‐ lesions, the performances of the models were not significantly different (See Supporting information Figure S4.) The difference in results may be due to (1) the major decrease in the number of the patients (96 vs 56) or (2) that the information about the presence or absence of rim+ lesions in a region's WM is driving the differences in the rim+/‐ models for the larger set of 96 individuals.

PwMS with greater disability had significantly more rim‐ lesions than those with lower disability, while there were no differences in the number of rim+ lesions between the disability groups. However, the model based on structural disconnection from rim+ lesions better predicted greater disability than the one based on structural disconnection from rim‐ lesions. This result supports the notion that greater disability does not directly track with the number of lesions, but rather, more likely, the size and/or location of the lesions and how they can disrupt structural connectivity networks. The previous studies showed that even though the correlation between lesion load and disability is weak (Miki et al., 1999), the lesion location can influence various clinical deficits, including motor dysfunction, as assessed by EDSS (Charil et al., 2003; Krieger & Lublin, 2018). Larger size of critically located lesions might also cause a greater disability.

The focus of this work was on analyzing the differences in the relationship between disability and structural disconnection patterns from rim+ and rim‐ lesions, and, furthermore, how damage from the two lesion types to certain regions or pairwise connections were informative of disability. We did not set out to identify the most accurate classification model; with the limited number of individuals in the study and large dimensionality of the imaging variables, a model with fewer variables likely will have higher AUC. In fact, a simple logistic regression model (cross‐validated in the same way as the ADA model) using only demographics (age, sex, race), clinical information (disease duration and treatment duration), and total *T*2 brain lesion volume had a median AUC of 0.72, which was significantly greater than the best demographics + imaging model (median AUC of 0.66). Future work focusing only on prediction accuracy (and not model inference, as we do here) using this data set may investigate dimensionality reduction techniques applied to high dimensional imaging measures to improve classification performance.

### 4.3 Disconnection in the left thalamus and left cerebellum are central to accurate disability classification

Examination of the feature importance values from the classification models revealed the central role of structural disconnectivity in the left thalamus and left cerebellum. Interestingly, these same regions were important regardless of the lesion type (rim+ or rim‐) causing disconnectivity. The thalamus plays an important role in a wide range of functions, such as cognition, memory, executive function, and motor ability (Alexander et al., 1986; Batista et al., 2012; Henry et al., 2008), and is known to be among the most affected regions in pwMS (Vercellino et al., 2009). Functional connectivity changes and structural changes in the thalamus, observed with anatomical and diffusion MRI, have been related to cognitive and motor impairment (Henry et al., 2008; Schoonheim et al., 2015; Tovar‐Moll et al., 2009). Previous work has shown the thalamus to be one of the only regions exhibiting a significant relationship between atrophy and structural disconnection in pwMS, which indicates this region may be particularly vulnerable to increased atrophy when lesions occur in its connecting WM (Kuceyeski et al., 2015). In addition, it has been shown that more thalamic atrophy was significantly related to increased EDSS in pwMS (Tao et al., 2009; Tovar‐Moll et al., 2009) and that pwMS with greater disability had significantly lower thalamic volume compared to healthy controls, while no differences were found between nonimpaired pwMS and controls (Zhong et al., 2017). Taken together, these studies indicate the central role of the thalamus in the development of greater disability in pwMS.

Many studies have shown relationships between cerebellar pathology and impairments in motor control and cognition (D'ambrosio et al., 2017; Weier et al., 2014). The presence of cerebellum‐related symptoms at the onset of MS, such as coordination issues or tremor, were (i) shown to be associated with shorter time to an EDSS of 6 (Weinshenker et al., 1991) and (ii) related to earlier onset of progressive disease diagnosis (Novotna et al., 2015). Atrophy in the anterior cerebellum was associated with motor dysfunction (D'ambrosio et al., 2017) and reduced total cerebellar volume was related to worse cognitive test performance (Weier et al., 2014) in pwMS. In our dataset, the patients with greater disability showed higher disconnectivity in the right and left cerebellum than the patients with lower disability.

### 4.4 Race, sex, age, and disease duration play a potentially important role in disability classification

In general, it seems the demographics had greater variable importance in the rim‐ model, which could be because the rim+ model which contains information about disability‐relevant rim+ status (since those without rim+ lesions have zero ChaCo scores in the rim+ model) dominates the classification and, therefore, the demographics have reduced importance. Race was one of the most important demographic predictors in the rim‐ model. It has been shown that African Americans (which make up the largest non‐Caucasian group in our study) generally have larger *T*1 and *T*2 lesion volumes, more severe disability at diagnosis and more severe disease progression (Cipriani & Klein, 2019; Weinstock‐Guttman et al., 2010; Weinstock‐Guttman et al., 2003). Sex also appeared to be an important predictor in the rim‐ lesion model for disability classification; specifically, being male was associated with higher probability of being in the greater disability group. It has been shown previously that male patients tend to have more severe disease onset with accelerated clinical progression in MS (Bove et al., 2012; Debouverie, Pittion‐Vouyovitch, Louis, & Guillemin, 2008; Gholipour, Healy, Baruch, Weiner, & Chitnis, 2011). Age and disease duration were also important features in the classification models; this is unsurprising as EDSS generally increases over the course of the disease.

#### 4.4.1 Limitations

The main limitation of our study is the sample size; classifiers are always more robust when they are trained and tested on larger datasets, particularly, when dealing with such a heterogenous disease as MS. The size of the dataset is equal to the number of input variables for the single‐modality rim+ and rim‐ ChaCo only models, but much less than the number of variables in the model including both modalities. This could explain the combined model's lower AUC compared to the rim+ only model. The moderate sample size was another reason we did not investigate pairwise structural disconnectivity matrices, as the number of variables would be much larger than the data in that case. Another drawback was the use of the NeMo tool, which estimates a lesion's structural disconnectivity based on a database of healthy controls that may not perfectly reflect the particular individual's structural connectivity network, particularly if there are pathologies in WM of pwMS that are not reflected in the lesion masks. However, MS lesions do disrupt diffusion MRI signals and can add noise to tractography results, so the NeMo Tool may be a good alternative with which to estimate structural disconnection. Another limitation is that the models used in our study considered only the lesion size and location and their subsequent regional structural disconnection. A future study may consider the impact of the severity and type of tissue damage within the different lesions on structural disconnectivity and subsequent disability. Additionally, while gray matter atrophy has been shown to be associated with disease progression in pwMS (Fisher et al., 2008), the aim of this work is not the most accurate classifier but to compare the accuracy of models based on structural disconnection from two different types of WM lesions (rim+ and rim‐). Another limitation is that spinal cord lesion information (number and size), or spinal cord atrophy which can be confounder variables were not used in our study. Chronic black holes, a conventional measure found to be associated with more axonal loss within lesions, were not considered in this analysis and can be considered for future expansion of this work. While our models included treatment duration, we did not include treatment type which can also differentially impact disability. Future work aiming to create the most accurate model classifying impairment in pwMS may also consider other imaging, treatment information, or demographic variables. Another limitation of our study was that the maps of rim‐ and rim+ lesions were not quantitatively compared using a statistical approach, but only qualitatively assessed—the large discrepancy of number of rim+ versus rim‐ lesions complicates both types of comparisons. The two lesion types do seem to be more centered around the periventricular space and the larger brain area seemingly impacted by rim‐ lesions may be a byproduct of their higher occurrence rate. Finally, this work only explored cross‐sectional relationships; future work should work to predict the probability of disease progression over time for use in clinical care decisions.

## 5 CONCLUSIONS

This work represents, to the best of our knowledge, the first to quantify and examine the differential impact of QSM rim+ and rim‐ lesions on the structural connectome and, furthermore, to use these measures of disconnectivity to classify pwMS into disability categories. Structural disconnectivity associated with rim+ lesions on QSM was slightly more related to greater disability than structural disconnectivity associated with rim‐ lesions. Damages to the structural connections of the left cerebellum and thalamus from either lesion type were especially impactful on greater disability. This analysis provides a deeper understanding of how different lesion types can disrupt the structural connectome and contribute to MS‐related disability. Deeper understanding of the role of the connectome in MS is needed if we need a comprehensive view of the disease to ultimately improve clinical outcomes in pwMS.

## AUTHOR CONTRIBUTION

C.T. designed the study, helped with processing the MRI data, carried out the statistical and machine learning analyses, drafted and wrote the manuscript.

K.J. developed the version 2.0 of Network Modification tool and edited the manuscript.

N.Z. collected and merged data.

T.N. assisted with image data processing and edited the manuscript.

S.P. performed data processing and edited the manuscript.

Y.W. assisted with data acquisition and analyses and edited the manuscript.

S.G. collected the data, supervised the analyses, helped interpret results, and edited the manuscript.

A.K. designed the study, supervised the analyses, and edited the manuscript.

## CONFLICT OF INTEREST

YW owns equity of Medimagemetric LLC. The authors declare that they have no other conflict of interest.

### PEER REVIEW

The peer review history for this article is available at https://publons.com/publon/10.1002/brb3.2353.

## CITATION GENDER DIVERSITY STATEMENT

We used classification of gender based on the first names of the first and last authors (Dworkin et al., 2020), with possible combinations including male/male, male/female, female/male, and female/female. The gender balance of papers cited within this work was quantified using gender‐api.com. The authors with a gender estimation accuracy lower than 90% were checked using manual gender determination from authors’ publicly available pronouns. Among the 59 cited works, 1 article had only one author. Among the 58 cited works with more than one author, 53% (n = 31) were MM, 21% (n = 12) were WM, 19% (n = 11) were MW, and 7% (n = 4) were WW.

## Supporting information

SUPPORTING INFORMATION

## Data Availability

The structural disconnectivity scores that support the findings of this study are available upon reasonable request. The codes that were used in this study are available at https://github.com/cerent/MS‐QSM.
