# Corrigendum

**DOI:** 10.1002/brb3.2480

**Published:** 2022-01-24

In the article by Tozlu et al. (2021) incorrect ChaCo scores were shown in Figures 3, 4, 5, and 6. The corrected figures appear below:

**FIGURE 3:**
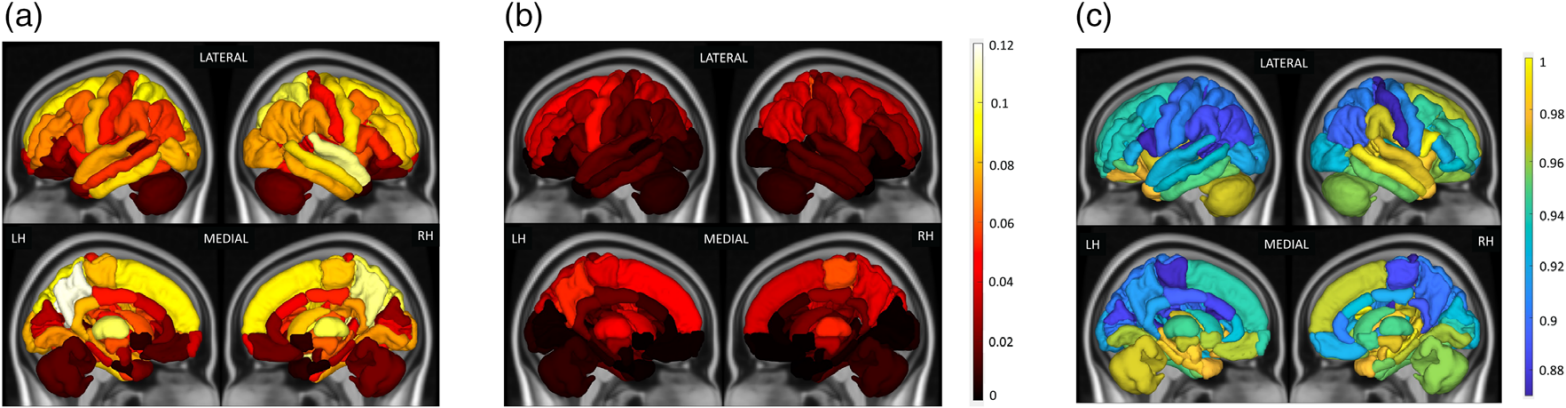
Median of ChaCo extracted from (A) rim‐ lesion mask (T2 FLAIR lesions excluding rim+ lesions) across all pwMS (N = 96) and (B) rim+ lesion masks, only for the pwMS who had at least one rim+ lesion (N = 56). The colorbar shows the ChaCo for the Figure (A) and (B). (C) Relative paired Wilcoxon rank‐sum statistic (divided by maximum value) indicating all regions had greater ChaCo from rim‐ lesion masks than from rim+ lesion masks (considering only the 56 pwMS who had at least one rim+ lesion). Median ChaCo from rim+ lesion masks were computed only for the subjects who had rim+ lesions (N = 56), while the median ChaCo from the rim‐ lesion mask was computed across all subjects. Note the scale differences in the two modalities–this is mostly due to the fact that there were far fewer rim+ lesions than rim‐ lesions.

**FIGURE 4:**
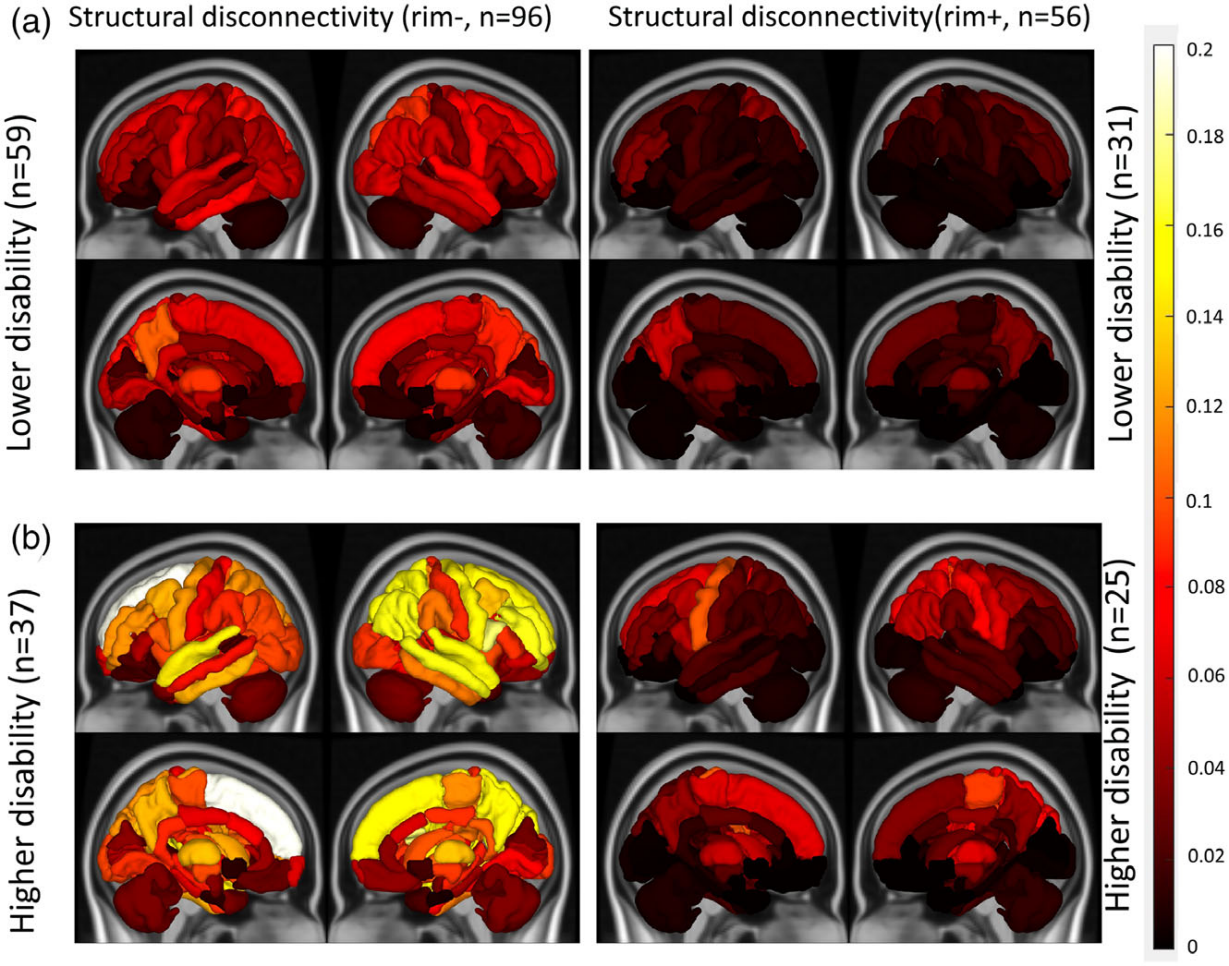
Median of ChaCo extracted from rim‐ (T2 FLAIR lesions excluding rim+ lesions) and rim+ lesion masks for pwMS (A) lower disability versus (B) those with greater disability

**FIGURE 5:**
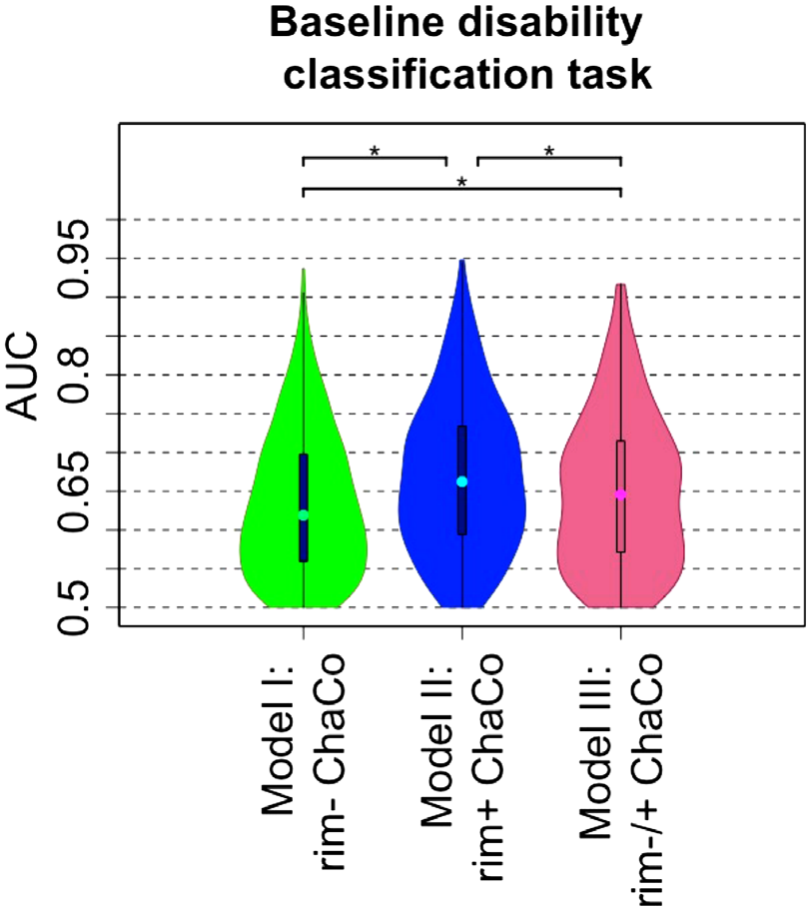
AUC results obtained with Model I (ChaCo from rim‐ lesion masks), Model II (ChaCo from rim+ lesion masks), and Model III (both rim‐ and rim+ lesion ChaCo) for the classification task of lower versus greater disability. AUC results were obtained over the 100 outer loops and 5 test datasets for each outer loop for the disability classification task. * indicates significant differences in AUC, corrected p < .05

**FIGURE 6:**
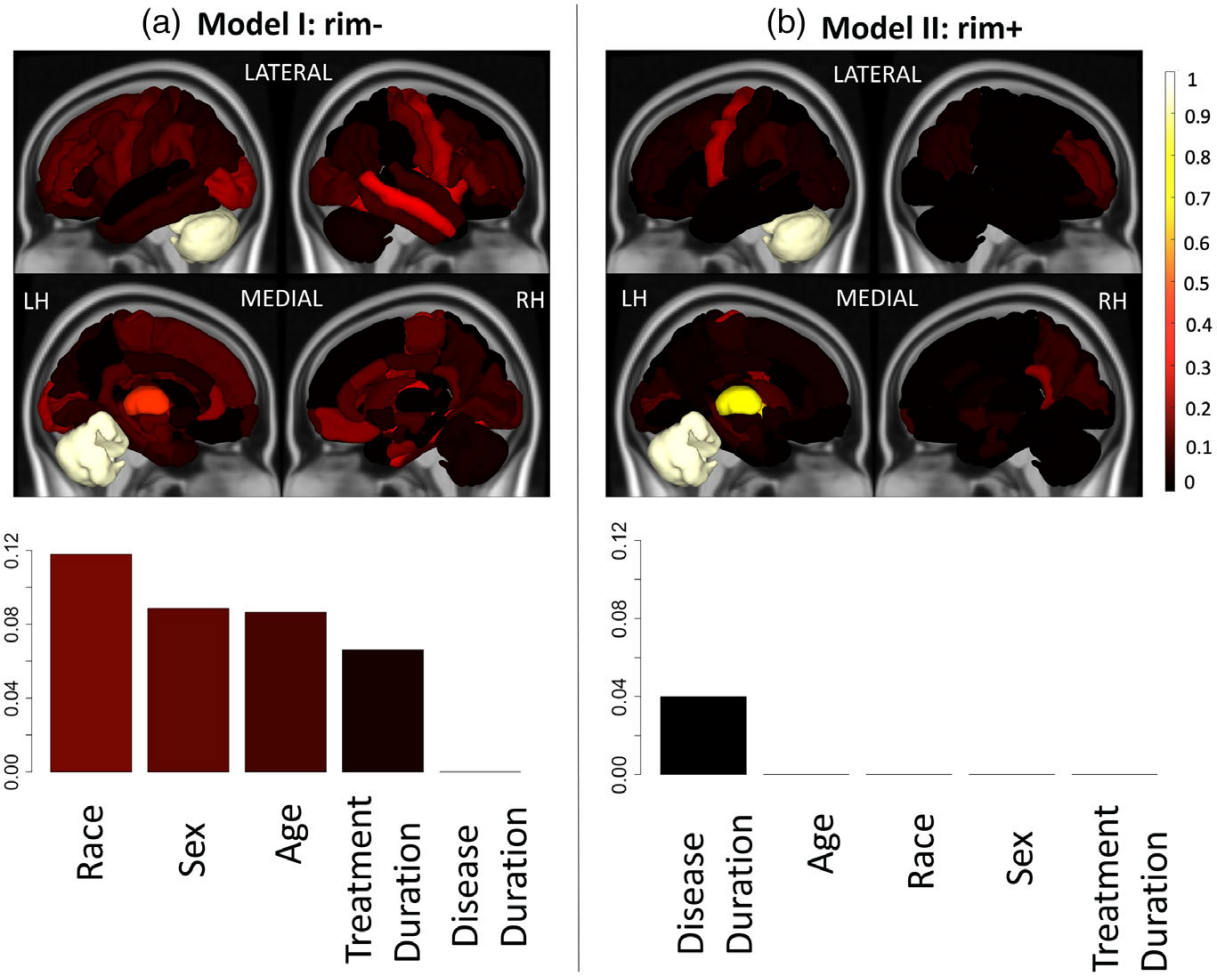
Relative feature importance for the models that included demographics and regional ChaCo due to (A) Model I: rim‐ lesions (T2 FLAIR lesions excluding rim+ lesions) (left column) and (B) Model II: rim+ lesions (right column) for the classification of pwMS with greater disability versus those with lower disability. Feature importance for the regional ChaCo scores are visualized via brain volumes and demographic variable importance by bar plots. Third quantiles of the feature importance distributions are visualized due to the distribution skewness. Relative importance values for all figures were obtained by dividing that variable's feature importance by the maximum importance value across both models

The authors apologize for the error.

## References

[brb32480-bib-0001] Tozlu, C. , Jamison, K. , Nguyen, T. , Zinger, N. , Kaunzner, U. , Pandya, S. , Wang, Y. , Gauthier, S. , & Kuceyeski, A. (2021). Structural disconnectivity from paramagnetic rim lesions is related to disability in multiple sclerosis. Brain and Behavior, 11, 1–13. 10.1002/brb3.2353 PMC8553317 34498432

