# Supplementary material for: Structural disconnectivity from paramagnetic rim lesions is related to disability in multiple sclerosis
Source: Brain Behav. 2021 Sep 8;11(10):e2353. doi: 10.1002/brb3.2353 (PMC8553317; doi:10.1002/brb3.2353)
Supplement: Supplementary file 1 — SUPPORTING INFORMATION [file BRB3-11-e2353-s001.docx]

**SUPPLEMENTARY MATERIAL**

**Title:** Structural disconnectivity from paramagnetic rim lesions is related to disability in multiple sclerosis

**Running title:** Disability classification using paramagnetic rim lesions in MS

**NeMo Tool**

The Network Modification 2.0 (NeMo 2.0) tool estimates structural connectivity disruption due to a lesion or injury from a database of healthy structural connectivity. We first computed a database of whole-brain tractograms for 420 unrelated subjects (206 female, 214 male, 28.7 ± 3.7 years) from the Human Connectome Project Young Adult (HCP-YA) dataset. The HCP diffusion data consists of 1.25mm isotropic voxels, 3 shells (b=1000,2000,3000) and 90 directions per shell, collected with both R-L and L-R phase encoding. HCP data have been minimally preprocessed to correct for motion, EPI and eddy-current distortion, and registered to subject T1 anatomy (M. F. Glasser et al., 2013). We used MRtrix3 to estimate a voxel-wise multi-shell, multi-tissue constrained spherical deconvolution (CSD) model (Jeurissen, Tournier, Dhollander, Connelly, & Sijbers, 2014), followed by whole-brain probabilistic tractography (iFOD2 (J Donald Tournier, Calamante, & Connelly, 2010) with anatomically constrained tractography – ACT (Smith, Tournier, Calamante, & Connelly, 2012)) using dynamic seeding to produce 5 million streamlines per subject. We also computed streamline weights to reduce known biases in tractography algorithms and better match the whole brain weighted tractogram to diffusion properties of the observed data (SIFT2, (Smith, Tournier, Calamante, & Connelly, 2015)). Streamlines for each HCP subject were warped into a common volumetric space (MNI152).

    Given a lesion mask in MNI space, the NeMo tool identifies the gray matter endpoints of all streamlines that pass through the masked voxels, and computes a regional structural disconnectivity (SDC) score for each gray matter region in a given atlas, representing the fraction of streamlines connecting to that gray matter region that have passed through the lesion. Disconnectivity is connected for each region for each of the 420 HCP database subjects, and then averaged across database subjects to create the final group SDC estimate for each region. A region with a SDC score of 0 is expected to have normal structural connectivity, while a SDC score of 1 suggests complete disconnection.

**Adaptive Boosting method and the parameters used in this study**

Adaptive Boosting (ADA) consecutively applies decision trees that split the data into two classes successively based on a randomly chosen variable at each node. Decision trees in ADA chose the variable which minimizes the Gini Index (GI) in the classification analysis. GI at node *t* is defined as

$$\sum_{c=1}^{L} \hat{p_{t^{c}}}(1-\hat{p_{t^{c}}})$$

where $\hat{p_{t^{c}}}$ is the proportion of the observation in class *c* at node *t*.

*adabag* library and *boosting* function in R version 3.4.4 were used for the ADA model creation. The minimum number of observations that must exist in a node in order for a split to be attempted (*minsplit*) and complexity parameter (*cp*) were optimized in the inner loop of cross-validation. The default value of *minsplit* and *cp* parameters are 20 and 0.01 in rpart.control function of R (1). To find the optimal value of these parameters for different train datasets, a grid search was applied for *minsplit* in the interval [5, 50with steps of 5 and for *cp* in the interval [0.001, 0.1by increments of 10. The pair of *minsplit* and *cp* parameters that maximized AUC in the inner loop was identified as the optimal pair of hyperparameters and then used to build the model. For each model, 50 decision trees were used.

The inner loop performed grid-search to find the set of hyperparameters that maximized area under the Receiver Operating Characteristics curve (AUC) in the validation set. Synthetic Majority Over-sampling Technique (SMOTE) (2) was used to obtain a class-balanced training dataset to improve the prediction accuracy for the minority class. SMOTE compensates for imbalanced classes by creating synthetic examples using nearest neighbor information instead of creating copies from the minority class, and has been shown to be among the most robust and accurate methods with which to control for imbalanced data (3). The inputs are standardized in the inner loop and in the outer loop to avoid data-leakage. A final model built using the entire training dataset with the optimal hyperparameters and assessed on the hold-out test set from the outer loop. The relative importance of the input variables in the final ADA models was calculated using the weight of the tree and gain of the Gini Index, which is given by a variable in a tree (4).

**References:**

1. Breiman L, Friedman J, J.Stone C, Olshen RA. Classification Algorithms and Regression Trees [Internet]. Mathematics- Taylor&Francis. 1984. 368 pages. Available from: https://rafalab.github.io/pages/649/section-11.pdf

2. Chawla N V., Bowyer KW, Hall LO, Kegelmeyer WP. SMOTE: Synthetic Minority Over-sampling Technique. J Artif Intell Res [Internet]. 2002 Jun 1 [cited 2019 May 29];16:321–57. Available from: https://jair.org/index.php/jair/article/view/10302

3. Santos MS, Soares JP, Abreu PH, Araujo H, Santos J. Cross-Validation for Imbalanced Datasets: Avoiding Overoptimistic and Overfitting Approaches [Research Frontier]. IEEE Comput Intell Mag [Internet]. 2018 Nov [cited 2019 Jul 22];13(4):59–76. Available from: https://ieeexplore.ieee.org/document/8492368/

4. Alfaro E, Gáamez M, García N. Adabag: An R package for classification with boosting and bagging. J Stat Softw [Internet]. 2013 Sep 3 [cited 2020 Jul 12];54(2):1–35. Available from: https://www.jstatsoft.org/index.php/jss/article/view/v054i02/adabag_An_R_Package_for_Classification_with_Boosting_and_Bagging.pdf

**Supplementary Table 1:** Number of pwMS with and without rim+ lesions divided by disability group.

|  | **No rim+ lesions** | **At least one rim+ lesion** | **Total** |
| --- | --- | --- | --- |
| **No disability** | 27 (67%) | 32 (57%) | 59 (61%) |
| **Disability** | 13 (33%) | 24 (43%) | 37 (39%) |
| **Total** | **40 (100%)** | **56 (100%)** | **96 (100%)** |

**Supplementary Table 2:**  Demographics and clinical variables for the pwMS without rim+ and with at least one rim+ lesion. The p-values that were obtained in comparison of groups were presented in the fourth column. Values are presented as median [1^st^ quartile, 3^rd^ quartile] for the continuous variables, p-values are corrected with Benjamini-Hochberg method for multiple comparisons. Age, disease and treatment duration were measured in years.

|  | **No rim+ lesions**  **(n=40)** | **At least one rim+ lesion**  **(n=56)** | **p-value** |
| --- | --- | --- | --- |
| **Age** | 45 [34.75, 49] | 37 [30.75, 47.25] | 0.11 |
| **Disease Duration** | 4.77 [2.75, 10.89] | 4.68 [2.44, 12.27] | 0.97 |
| **Treatment Duration** | 3.23 [1.73, 5.34] | 3.16 [1.36, 7.06] | 0.97 |
| **EDSS** | 1 [0,2] | 1.5 [0, 2] | 0.34 |
| **Female (%)** | 26 (46%) | 38 (68%) | 0.94 |
| **Race** | African American: 6 Asian: 1  Caucasian: 31  Hispanic: 2  Other: 0 | African American: 10  Asian: 1  Caucasian: 38  Hispanic: 2  Other: 5 | 0.38 |
| **Total lesion volume (ml)** | 82.08 [35.11, 208.50] | 83.08 [43.51, 294.05] | 0.44 |


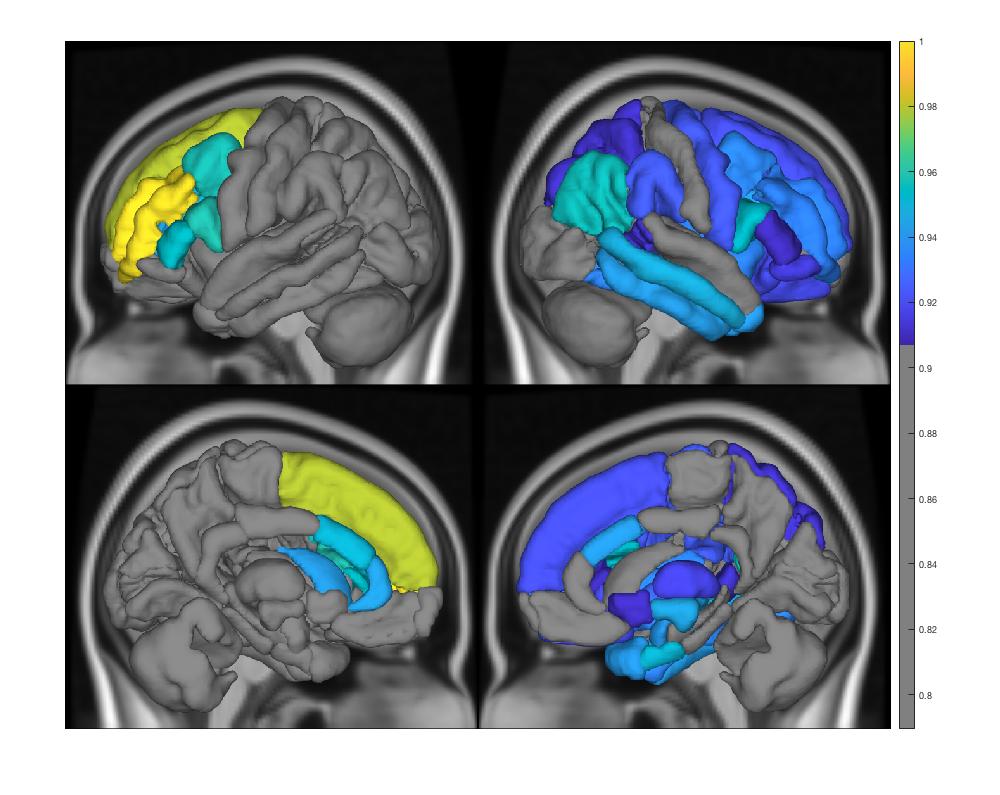


**Supplementary Figure 1:** The gray matter regions that had significantly greater (corrected p < 0.05) structural disconnection due to rim- lesions in pwMS that had greater disability compared to those with lower disability. The color bar represents the relative group comparison statistic obtained from Wilcoxon rank sum test.

**Supplementary Figure 2:** Classification results (sensitivity, specificity, and balanced accuracy) in distinguishing pwMS according to disability group**.** *indicates a significant difference in that metric between pairs of models, corrected p < 0.05.


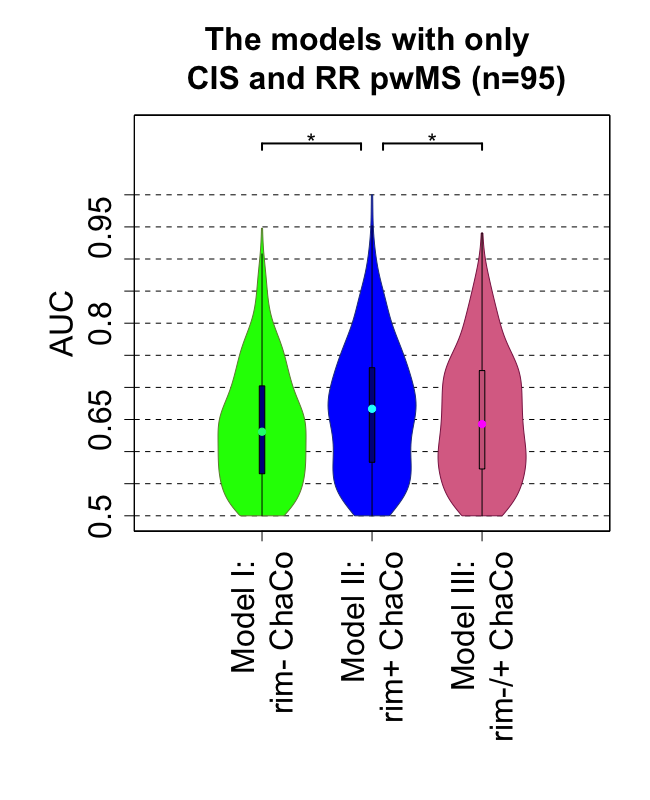


**Supplementary Figure 3:** The AUC results in distinguishing pwMS according to disability group when only CIS and RRMS patients were used (i.e. one pwMS who had PPMS was excluded) (n=95)**.** *indicates a significant difference in that metric between pairs of models, corrected p < 0.05.

**
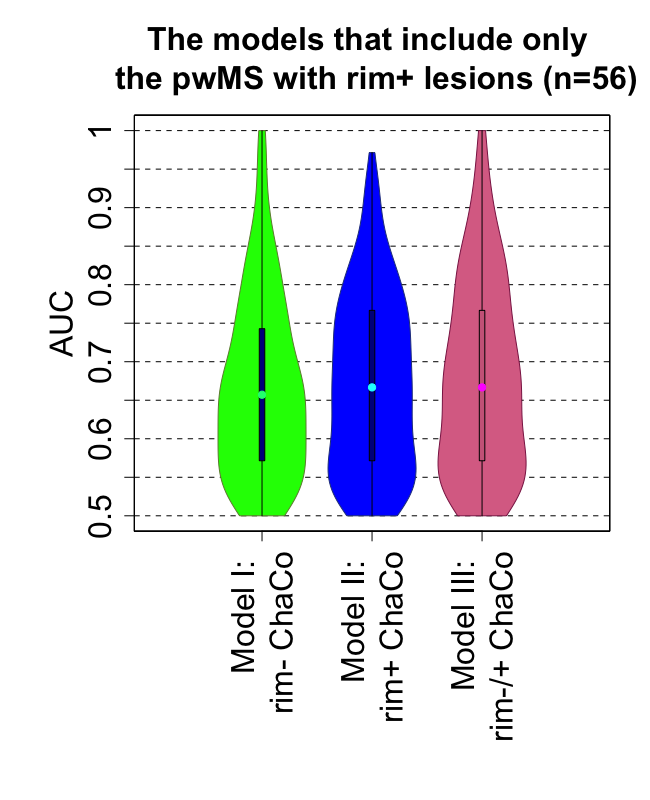
**

**Supplementary Figure 4:** The AUC results in distinguishing pwMS according to disability group when only the pwMS who had rim+ lesions were used (n=56)**.** *indicates a significant difference in that metric between pairs of models, corrected p < 0.05.

**Supplementary Table 3:** Relative feature importance for the models that included demographics and regional ChaCo due to (A) Model I: rim- lesions (T2 FLAIR lesions excluding rim+ lesions) and (B) Model II: rim+ lesions for the classification of pwMS with greater disability vs those with lower disability. Third quantiles of the feature importance distributions are computed due to the distribution skewness. Relative importance values were obtained by dividing that variable’s feature importance by the maximum importance value across both models.

| **Model I: rim- model** | | **Model II: rim+ model** | |
| --- | --- | --- | --- |
| **Relative Importance** | **Variable** | **Relative Importance** | **Variable** |
| 1.00000000 | lh -Cerebellum-Cortex | 1.00000000 | lh-Cerebellum-Cortex |
| 0.37848534 | lh -Thalamus-Proper | 0.74899683 | lh-Thalamus-Proper |
| 0.29554275 | rh-middletemporal | 0.64663507 | lh-Pallidum |
| 0.29397334 | rh-precentral | 0.23288851 | lh-precentral |
| 0.26905206 | rh-insula | 0.15133741 | rh-isthmuscingulate |
| 0.26895321 | lh-rostralanteriorcingulate | 0.12513735 | lh-pericalcarine |
| 0.22519740 | lh-lateraloccipital | 0.12029313 | lh-Putamen |
| 0.19510795 | lh -Pallidum | 0.11768785 | lh-Hippocampus |
| 0.18579355 | lh-rostralmiddlefrontal | 0.10980343 | rh-cuneus |
| 0.17493910 | lh-superiorfrontal | 0.10603703 | rh-pericalcarine |
| 0.16616049 | rh-fusiform | 0.10003911 | lh-Caudate |
| 0.16570895 | rh-medialorbitofrontal | 0.09058788 | rh-rostralmiddlefrontal |
| 0.15727370 | lh -Amygdala | 0.07955145 | rh-precuneus |
| 0.15596622 | lh-pericalcarine | 0.07523439 | lh-lateraloccipital |
| 0.15038379 | rh-lingual | 0.06471349 | lh-supramarginal |
| 0.14388046 | rh-parstriangularis | 0.06196009 | rh-frontalpole |
| 0.14372900 | Race | 0.06100957 | lh-parahippocampal |
| 0.13816874 | rh-isthmuscingulate | 0.06009711 | lh-Amygdala |
| 0.13720601 | rh-caudalanteriorcingulate | 0.05412542 | lh-caudalmiddlefrontal |
| 0.13622347 | rh-entorhinal | 0.05080061 | lh-temporalpole |
| 0.12865889 | rh-Caudate | 0.04901209 | lh-cuneus |
| 0.11722271 | lh-paracentral | 0.04871557 | Disease Duration |
| 0.11414393 | rh-parsopercularis | 0.04742153 | rh-Accumbens-area |
| 0.11190677 | lh-temporalpole | 0.04645938 | lh-rostralmiddlefrontal |
| 0.11038868 | lh-postcentral | 0.04382332 | lh-posteriorcingulate |
| 0.10991326 | lh-isthmuscingulate | 0.04294926 | lh-entorhinal |
| 0.10936625 | rh-rostralanteriorcingulate | 0.04209608 | rh-Amygdala |
| 0.10397291 | rh-Pallidum | 0.04201024 | rh-parsorbitalis |
| 0.10376950 | lh-precentral | 0.04196140 | rh-VentralDC |
| 0.10120893 | rh-inferiortemporal | 0.03912838 | rh-inferiorparietal |
| 0.10116432 | lh-parahippocampal | 0.03853165 | lh-superiorparietal |
| 0.10066413 | rh-VentralDC | 0.03431878 | lh-lateralorbitofrontal |
| 0.09979800 | rh-postcentral | 0.03349929 | lh-paracentral |
| 0.09812921 | lh-parsorbitalis | 0.03334935 | lh-inferiorparietal |
| 0.09749726 | lh-parsopercularis | 0.03152227 | lh-Accumbens-area |
| 0.09693082 | rh-cuneus | 0.03143381 | rh-superiorparietal |
| 0.09250668 | lh-Hippocampus | 0.03082671 | lh-VentralDC |
| 0.09087179 | rh-lateraloccipital | 0.02781129 | lh-bankssts |
| 0.09062715 | lh-insula | 0.02758141 | rh-insula |
| 0.08866281 | rh-frontalpole | 0.02655171 | rh-entorhinal |
| 0.08813058 | Age | 0.02525260 | rh-caudalanteriorcingulate |
| 0.08812129 | rh-paracentral | 0.02503564 | lh-rostralanteriorcingulate |
| 0.08730370 | rh-rostralmiddlefrontal | 0.02346475 | rh-Caudate |
| 0.08492648 | Gender | 0.01820412 | lh-superiorfrontal |
| 0.08484633 | lh-supramarginal | 0.01544513 | rh-Putamen |
| 0.08434413 | rh-Putamen | 0.01511313 | lh-insula |
| 0.08038201 | lh-entorhinal | 0.01269112 | rh-Pallidum |
| 0.07876519 | lh-VentralDC | 0.00000000 | rh-Cerebellum-Cortex |
| 0.07810581 | lh-caudalmiddlefrontal | 0.00000000 | rh-Thalamus-Proper |
| 0.07103655 | lh-bankssts | 0.00000000 | rh-Hippocampus |
| 0.07092974 | lh-superiortemporal | 0.00000000 | lh-caudalanteriorcingulate |
| 0.07009439 | rh-inferiorparietal | 0.00000000 | lh-fusiform |
| 0.06899607 | Treatment Duration | 0.00000000 | lh-inferiortemporal |
| 0.06591604 | rh-caudalmiddlefrontal | 0.00000000 | lh-isthmuscingulate |
| 0.06591488 | rh-precuneus | 0.00000000 | lh-lingual |
| 0.06494562 | lh-fusiform | 0.00000000 | lh-medialorbitofrontal |
| 0.06461238 | lh-lateralorbitofrontal | 0.00000000 | lh-middletemporal |
| 0.06320234 | rh-superiortemporal | 0.00000000 | lh-parsopercularis |
| 0.06082081 | rh-supramarginal | 0.00000000 | lh-parsorbitalis |
| 0.05834895 | lh-caudalanteriorcingulate | 0.00000000 | lh-parstriangularis |
| 0.05765921 | lh-Putamen | 0.00000000 | lh-postcentral |
| 0.05658051 | lh -Accumbens-area | 0.00000000 | lh-precuneus |
| 0.05590501 | rh -Amygdala | 0.00000000 | lh-superiortemporal |
| 0.05579747 | rh-parsorbitalis | 0.00000000 | lh-frontalpole |
| 0.05502557 | lh-inferiortemporal | 0.00000000 | lh-transversetemporal |
| 0.05383003 | rh -Thalamus-Proper | 0.00000000 | rh-bankssts |
| 0.05241286 | rh-pericalcarine | 0.00000000 | rh-caudalmiddlefrontal |
| 0.05094241 | lh-posteriorcingulate | 0.00000000 | rh-fusiform |
| 0.04972318 | rh-posteriorcingulate | 0.00000000 | rh-inferiortemporal |
| 0.04676374 | rh -Accumbens-area | 0.00000000 | rh-lateraloccipital |
| 0.04109179 | rh -Hippocampus | 0.00000000 | rh-lateralorbitofrontal |
| 0.04055435 | Disease Duration | 0.00000000 | rh-lingual |
| 0.03818915 | lh-parstriangularis | 0.00000000 | rh-medialorbitofrontal |
| 0.03815252 | lh-middletemporal | 0.00000000 | rh-middletemporal |
| 0.03425053 | lh-frontalpole | 0.00000000 | rh-parahippocampal |
| 0.03281668 | lh-inferiorparietal | 0.00000000 | rh-paracentral |
| 0.03132442 | lh-superiorparietal | 0.00000000 | rh-parsopercularis |
| 0.02943737 | lh-precuneus | 0.00000000 | rh-parstriangularis |
| 0.02386422 | lh-cuneus | 0.00000000 | rh-postcentral |
| 0.00000000 | lh -Caudate | 0.00000000 | rh-posteriorcingulate |
| 0.00000000 | rh -Cerebellum-Cortex | 0.00000000 | rh-precentral |
| 0.00000000 | lh-lingual | 0.00000000 | rh-rostralanteriorcingulate |
| 0.00000000 | lh-medialorbitofrontal | 0.00000000 | rh-superiorfrontal |
| 0.00000000 | lh-transversetemporal | 0.00000000 | rh-superiortemporal |
| 0.00000000 | rh-bankssts | 0.00000000 | rh-supramarginal |
| 0.00000000 | rh-lateralorbitofrontal | 0.00000000 | rh-temporalpole |
| 0.00000000 | rh-parahippocampal | 0.00000000 | rh-transversetemporal |
| 0.00000000 | rh-superiorfrontal | 0.00000000 | Age |
| 0.00000000 | rh-superiorparietal | 0.00000000 | Gender |
| 0.00000000 | rh-temporalpole | 0.00000000 | Race |
| 0.00000000 | rh-transversetemporal | 0.00000000 | Treatment Duration |
